# Neural Plasticity Induced by Working Memory Training: Insights From Cortical Microstructure and Transcriptional Profiles

**DOI:** 10.1111/cns.70479

**Published:** 2025-07-30

**Authors:** Tian Zhang, Yuntao Gao, Yijun Li, Lin Wu, Xinxin Lin, Yilin Hou, Wei He, Yuanqiang Zhu, Jun Jiang, Yuanjun Xie, Peng Fang

**Affiliations:** ^1^ School of Military Medical Psychology The Fourth Military Medical University Xi'an China; ^2^ Department of Radiation Protection Medicine, Department of Military Preventive Medicine Air Force Military Medical University Xi'an China; ^3^ Department of Radiology Xijing Hospital, Fourth Military Medical University Xi'an China; ^4^ The Youth Innovation Team of Shaanxi University, Equipment Management and Unmanned Aerial Vehicle Engineering School, Air Force Engineering University (AFEU) Xi'an China; ^5^ Shaanxi Provincial Key Laboratory of Bioelectromagnetic Detection and Intelligent Perception Xi'an China; ^6^ Military Medical Innovation Center Fourth Military Medical University Xi'an China; ^7^ School of Biomedical Engineering Fourth Military Medical University Xi'an China

**Keywords:** gene expression, morphometric similarity network, neural plasticity, neuroanatomy, working memory training

## Abstract

**Aims:**

To investigate the effects of an 8‐week standardized computerized working memory training (WMT) program on cortical microstructure, morphometric similarity network (MSN) changes, and associated genetic factors in healthy adults.

**Methods:**

A total of 76 participants were divided into WMT and control groups. Cortical morphological measurements, including cortical thickness (CT) and fractional dimensions (FD), were measured. MSN changes based on CT and FD measures were analyzed. Additionally, partial least squares (PLS) analysis was conducted to investigate the relationship between critical microstructural alterations and gene transcript expression levels.

**Results:**

The WMT group exhibited reduced response times for updating, switching functions, and phonological loop tasks. The cortical morphological measurements revealed increases in CT in several right frontal regions, as well as FD in the frontopolar and middle frontal areas after WMT compared to baseline. Furthermore, significant decreases in MSN based on CT measures were found in specific occipital and intraparietal sulci. Similarly, the MSN of FD showed notable decreases in eigenvector and degree centrality in the left frontomarginal cortex and right middle temporal gyrus. PLS analysis revealed strong links between microstructural changes and gene expression, with PLS+ genes enriched in synaptic transmission, neural regulation, and energy metabolism, while PLS− genes were associated with intracellular transport, protein modification, and stress responses.

**Conclusion:**

The findings highlight the subtle influences of WMT on brain structure and underlying biological processes, providing insights into its role in neural plasticity and suggesting potential genetic contributions to these structural changes.

## Graphical abstract

An 8‐week WMT reduced the reaction time of the memory task and increased CT and FD in specific brain regions. MSN analysis of CT and FD reveals notable nodal topological changes in certain areas. Additionally, PLS analysis revealed strong links between WMT‐induced microstructural changes and gene transcript expression.

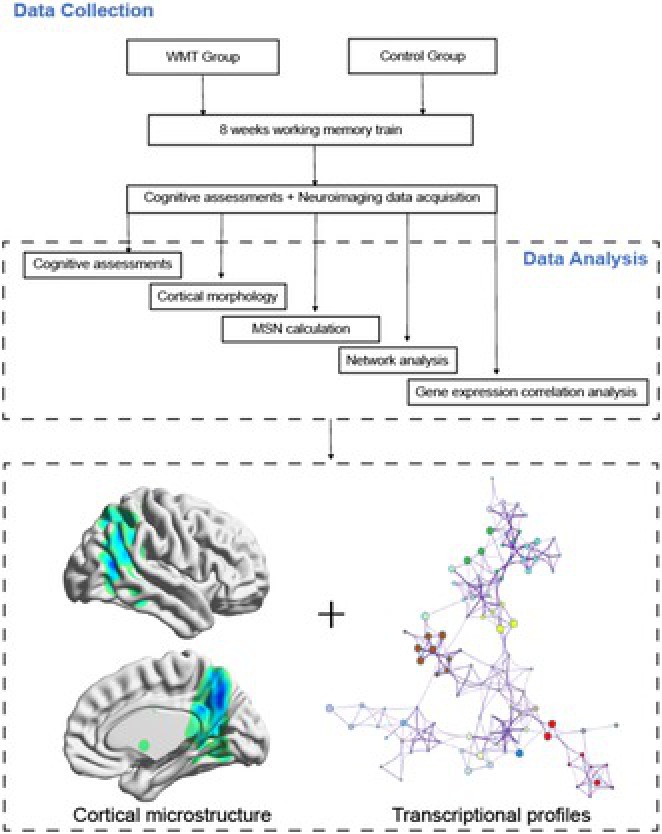

## 1 Introduction

Working memory (WM) is an essential cognitive process that allows for the temporary storage and manipulation of information [1]. As a result of its fundamental significance in overall cognition, WM has attracted increased attention in efforts to achieve widespread cognitive improvement through extended training on WM tasks [2]. Recent research has indicated that WM training (WMT) not only enhances WM capacity [3, 4], but also has positive impacts on general fluid intelligence and attentional control [5, 6].

To date, numerous studies have focused on the application of magnetic resonance imaging (MRI) for detecting specific architectural changes resulting from WMT. Following WMT, researchers have observed structural changes, including an increase in fractional anisotropy (FA) measures in the white matter regions adjacent to the intraparietal sulcus [7], as well as an increase in gray matter volumes in the right superior parietal cortex [8]. Nevertheless, the majority of the studies involved only single MRI morphometric and anatomical features, such as cortical thickness, volume, and curvature [9, 10], which may neglect individual differences and fail to reveal specific alterations in cortical morphology.

Single‐subject morphometric similar network (MSN) has been developed to construct morphological brain networks at the individual level [11, 12, 13]. Recent studies have begun to explore the topological characteristics in single‐subject MSN in the research of normal brain and neuropsychiatric disorders [14, 15, 16, 17, 18, 19, 20]. For instance, Seidlitz et al. demonstrated that MSN provides a novel, robust, and biologically plausible approach to understanding how human cortical networks underpin individual differences in psychological functions [21]. Similarly, patients diagnosed with major depressive disorder exhibited increased nodal centralities in parietal regions, while experiencing decreased nodal centralities in the temporal regions, as indicated by cortical thickness‐based MSN [22]. These results suggest that individual‐level single‐subject MSN could serve as a meaningful and reliable tool to explore the human connectome, but have yet to be tried in WMT.

Additionally, genetic factors play a significant role in shaping the brain's structure and functionality [23, 24]. Transcription‐neuroimaging association studies have established a strong correlation between gene expression profiles and various aspects of brain function and structure [25, 26, 27]. These include measures such as spontaneous brain activity (e.g., amplitude of low‐frequency fluctuations and regional homogeneity) [28, 29, 30], anatomical measures (e.g., gray matter volume and cortical thickness) [31, 32, 33, 34], connectivity [35, 36, 37], and MSN [38, 39, 40]. The found enriched genes are associated with nervous system development, synaptic transmission, and metabolic processes in the brain [41, 42, 43]. Additionally, the individual morphological similarity between cortical areas has been found to be aligned with spatial expression patterns of certain important genes [40]. Combining MSN and AHBA transcriptomic datasets would provide insight into how WMT‐related neuroplasticity varies at the microscale architecture.

The current study builds on prior WMT research by focusing on cortical microstructure and MSN, which have not been extensively explored in previous studies. While earlier research primarily examined single MRI morphometric features like cortical thickness and volume, this study introduces MSN to capture individual‐level morphological brain networks. Additionally, the study integrates gene expression data to explore the biological mechanisms underlying WMT‐induced neuroplasticity, which is a novel contribution to the field. The combination of MSN and transcriptomic analysis provides a more comprehensive understanding of how WMT influences brain structure and function at both macroscopic and microscopic levels. This study aims to investigate two primary hypotheses: (1) that WMT induces measurable changes in the cortical microstructure and topological properties of MSN, particularly in regions associated with working memory; and (2) that these structural changes are correlated with anatomically patterned gene expression, reflecting the genetic underpinnings of WMT‐induced neuroplasticity. To test these hypotheses, we employed Partial Least Squares (PLS) regression to analyze the relationship between brain structural indicators and region‐specific gene expression profiles. This integrative approach, which combines neuroimaging and genomic data, has been methodologically validated in prior research [44, 45], and is expected to provide novel insights into the mechanisms through which WMT influences brain structure and function at both the macroscopic and molecular levels.

## 2 Methods

### 2.1 Participants

**FIGURE 1:**
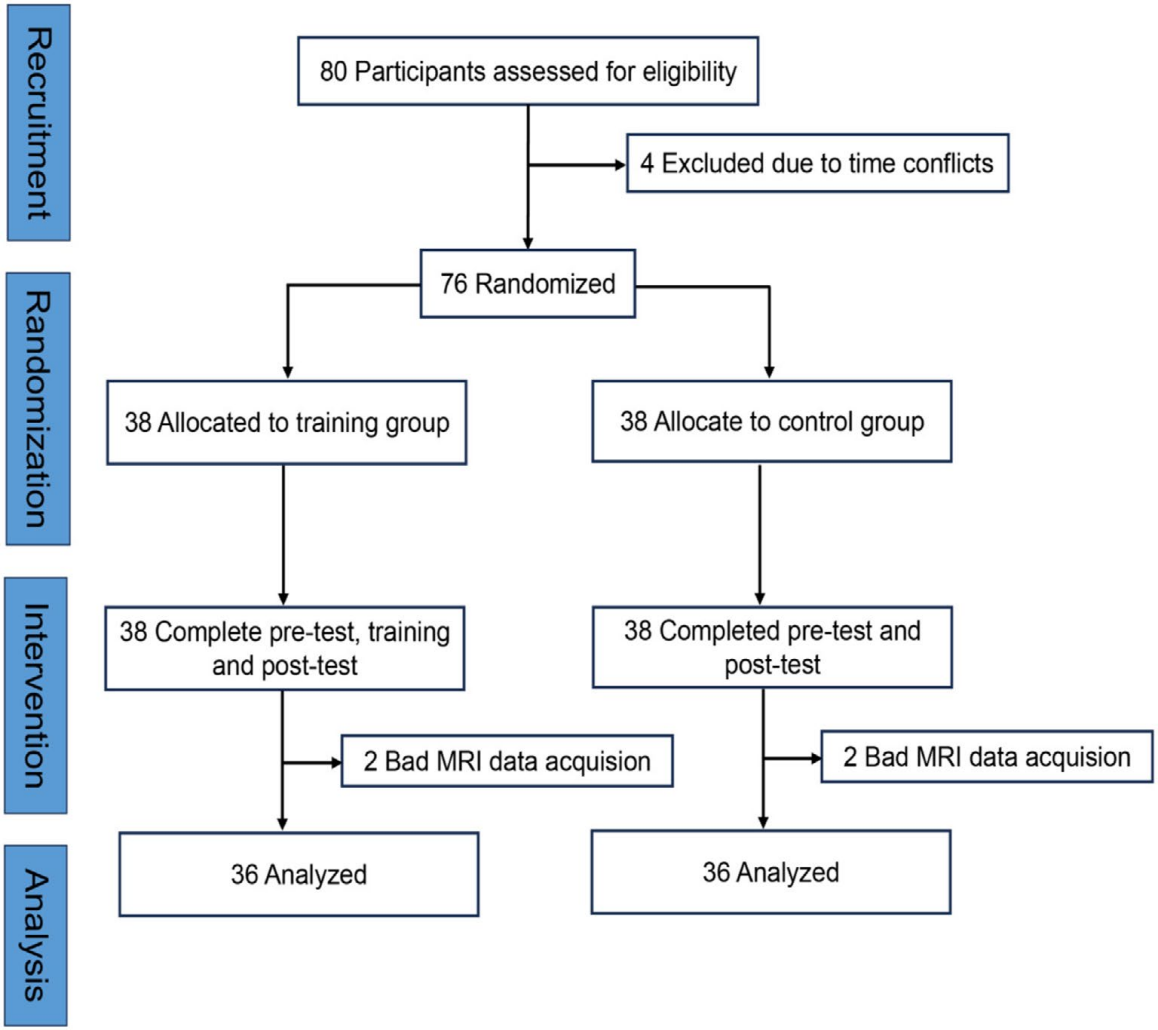
Flowchart of this study.

A total of 76 healthy participants were recruited for this study. They were equally divided into two groups: the WMT group and the control group, with each group consisting of 38 individuals. Two participants in the WMT group and two in the control group were excluded due to bad image quality. Finally, 36 participants from the WMT group and control group were included in this study, respectively (Figure 1). The sample size was calculated using G*Power software, with a statistical power (1‐*β*) of 0.90, an alpha level of 0.05, and a medium effect size (Cohen's *d* = 0.5). This calculation suggests that a total sample size of 36 would be sufficient to detect group differences in either matched pairs or independent tests. The following were the inclusion criteria: no prior medical treatment or drug use; normal eyesight without color blindness or weakness; no history of neurological or psychiatric diseases; and no use of alcohol, coffee, or other substances within 24 h of the pre‐ and post‐test. Individuals with any medical condition (such as pacemakers or certain metal implants) that might make an MRI scan inappropriate were not allowed to participate. Furthermore, participation was restricted to those who had already participated in other cognitive training programs or had a history of substance abuse.

### 2.2 WMT Protocol

#### 2.2.1 Running Memory Task

A computerized and standardized adaptive running memory task was used to perform WMT [46]. The task is commonly used to assess WM updating, which is a critical component of the central executive component of WM [47]. During the training assignments, a series of stimuli (locations, letters, and animals) were subsequently presented in the central region of a computer screen for a specific period (5, 7, 9, or 11). The participants were instructed to memorize the properties of the last three stimuli (e.g., animals) that appeared in each trial. They had to regularly update their memories, thereby engaging their WM. The training task consisted of 30 trials, broken into six segments of five trials each. Prior to the initiation of training, each item was displayed for a duration of 1750 ms. In the following block, the time was reduced by 100 ms for each participant who provided correct responses for three or more trials in a given block.

The length of the final segment a participant finished on a particular day determined the amount of training they received the following day. The participants were allocated an initial 30 min per day to complete the training, which was subsequently decreased to 20 min on the final day. The supervision of compliance with the training regimen was carried out using computerized WMT software, which recorded the training data of each participant. The training regimen consisted of 40 sessions per week over a period of 8 weeks, with five training days per week. The schedule included 3 days of exercise back‐to‐back, then 1 day of rest, 2 more days of exercise, and one final day of rest.

#### 2.2.2 Simple Memory Task

A simple memory task was allocated to the control group. Every participant was obligated to complete a 90‐trial memory task on each training day, which occurred 5 days a week. A 300 ms fixation point was consistently displayed in the center of the screen during the whole task. Subsequently, an animal appeared in the center of the screen for a duration of 1750 ms. After being shown nine different animals as options, participants were assigned the challenge of recalling which animal had been presented earlier. Participants were asked to finish it as quickly as possible. The participants allocated an average of 10 min each day to the training.

#### 2.2.3 Procedure

Upon providing demographic information and fulfilling online screening questionnaires, individuals who met the inclusion criteria were invited to the laboratory to do the pre‐test. The individuals who successfully completed the task were subsequently allocated at random to either the control or training cohort. Subsequently, during the course of the following 8 weeks, every participant attended the laboratory to carry out a total of 40 training sessions that aligned with their respective cohort. Participants returned at the end of the training session to complete the post‐test assignment. The theoretical foundation of the 8‐week training period has been supported by previous cognitive training research [48, 49, 50, 51].

### 2.3 Cognitive Assessments

#### 2.3.1 Evaluation of the Near‐Transfer Effect

There are three specific assessments of cognitive function: the update function test (UFT), the inhibition function test (IFT), and the switching function test (SFT). These tests are considered near‐transfer tests since they closely relate to WM. Essentially, UFT involves participants memorizing a sequence of letters shown on the screen and then deciding if a later set of three letters matches the ones they remembered. This assessment measures their proficiency in updating. The IFT entails a Stroop task that requires participants to determine the bigger numerical value between two Arabic numerals of varied sizes, with an emphasis on value rather than physical size. This task assesses their capacity to suppress automatic responses. The SFT evaluates the capacity to shift attention between tasks. Participants distinguish the significance of red numerals and the evenness or oddness of blue digits in a scenario involving two tasks. The task specifics were thoroughly explained in a prior investigation [52].

#### 2.3.2 Evaluation of the Far‐Transfer Effect

The far transfer effect tests, which include the visuospatial sketchpad test (VST) and the phonological loop test (PLT), are primarily intended to evaluate cognitive abilities associated with working memory training. The VST task requires participants to memorize the locations of black squares inside a 4 × 4 grid. Subsequently, they must determine if the final two squares match any of the previously displayed placements. This task assesses visuospatial memory and attention abilities. The phonological loop test (PLT) involves the retention of a series of capital letters and subsequently identifying whether a new lowercase letter matches any of the previous letters, hence assessing phonological processing ability. These specific tasks have been extensively described in previous studies [52].

### 2.4 Neuroimaging Data Acquisition and Processing

The high‐resolution 3D T1‐weighted structural MRI images were acquired by a 3.0 T scanner (GE Discovery MR 750, Milwaukee, Wisconsin, US). Specifically, the magnetization‐prepared rapid gradient‐echo sequence was used to obtain individual structural MRI images with the following parameters: repetition time = 8.1 ms; echo time = 3.2 ms; inversion time = 900 ms; flip angle = 10^0^; field of view = 240 × 240 mm; matrix size = 256 × 256 mm; voxel size = 1 × 1 × 1 mm, with no gap.

The CAT12 toolbox (http://www.neuro.uni‐jena.de/cat/) was utilized to process all structural MRI images, following the Statistical Parametric Mapping software (SPM12, http://www.fil.ion.ucl.ac.uk/spm/softwarespm12) framework. Briefly, the initial step involved segmenting individual structural pictures into distinct components: gray matter, white matter, and cerebrospinal fluid. Subsequently, the cortical thickness (CT) was estimated, and the central surface was reconstructed using a projection‐based thickness approach [53]. Furthermore, CAT12 enables the estimation of other morphological indices such as fractional dimension (FD), gyrification index (GI), and sulcus depth (SD). These indices were also computed for each participant using the default parameter settings. After getting the morphological maps, they were resampled onto the fsaverage template and smoothed with a Gaussian kernel that had a full width at half maximum (FWHM). More precisely, the CT maps of each individual were subjected to smoothing using a Gaussian kernel with a full width at half maximum (FWHM) of 15 mm. Similarly, additional surface maps, such as FD maps, were smoothed using a Gaussian kernel with an FWHM of 25 mm, as described by Ruan et al. [14]. The reason for using bigger filter sizes for the FD, GI, and SD maps is because these folding measures capture information from both sulci and gyri. Therefore, the filter size needs to be larger than the distance between a gyral crown and a sulcus.

### 2.5 Cortical MSN Calculation

#### 2.5.1 Definition of Network Nodes

The cortical MSN matrices were computed to evaluate the similarity in cortical patterns among various brain areas using different morphological indicators. Within the matrices, the nodes served as representations of brain regions. A well‐applied Destrieux surface atlas was used to define nodes [54]. This atlas divides the cerebral cortex into 148 regions. Each region represented a node in the morphological similarity matrices. The Destrieux atlas was chosen for studying nodal features because it has been shown to be a good balance between the amount of computing needed and the consistency of results when examining individual cortical MSN [14].

#### 2.5.2 Definition of Network Edges

Furthermore, the interregional similarity in the distribution of regional morphology was utilized to calculate the edges between the nodes. The edge estimations utilized the Jensen‐Shannon Divergence (JSD) approach, which was selected due to its ability to (1) quantify dissimilarity between probability distributions, (2) handle non‐linear relationships, and (3) avoid assumptions of normality. JSD is a type of Kullback–Leibler divergence (KLD) that quantifies the dissimilarity between two probability distributions. Initially, the values of all vertices within each node were extracted for each morphological index. Next, a probability density estimate was calculated for each node and morphological index by using a Gaussian kernel smoothing density function. To find the JSD, the probability density estimates were turned into probability distribution functions (PDFs). The KLDs between two PDFs *P* and *Q* are computed as:

$${\mathrm{KLD}}_{s}\left(P,Q\right)=e^{-D_{\mathrm{KL}}\left(P,Q\right)},$$

where *e* is the natural base and $\begin{array}{c} D_{\mathrm{KL}}\left(P,Q\right)=\mathrm{KLD}\left(P\|Q\right)+ \end{array}$ $\begin{array}{c} \mathrm{KLD}\left(P\|Q\right)=\sum_{i=1}^{n}P\left(i\right)\log\frac{P\left(i\right)}{Q\left(i\right)}+\sum_{i=1}^{n}Q\left(i\right)\log\frac{Q\left(i\right)}{P\left(i\right)} \end{array}$, with *n* represents the number of sampling points used in the probability density estimate. In this study, *n* is equal to 2^8^ for each node. The morphometric similarity (JSDs) was calculated by taking the square root of the JSD and then subtracting it from 1. It was defined as:

$${\mathrm{JSD}}_{s}\left(P,Q\right)=1-\sqrt{\mathrm{JSD}\left(P,\|,Q\right)},$$

Where $\text{JSDQ}\left(P\|Q\right)=\frac{1}{2}\mathrm{KLD}\left(P\|\frac{1}{2}\left(P+Q\right)\right)+\frac{1}{2}\mathrm{KLD}\left(Q\|\frac{1}{2}\left(P+Q\right)\right)$. Following the aforementioned methods, each participant yielded a total of four MSN matrices (148 × 148). The matrices had values representing the degree of similarity in morphology across distinct regions. This similarity ranged from 0 to 1, where a value of 0 indicated that two regional PDFs were completely different, and a value of 1 indicated that they were exactly the same.

### 2.6 Network Analysis

#### 2.6.1 Threshold Method

The matrices were converted to binary networks using a thresholding process based on sparsity. Sparsity is the measure of the density of edges in a network, calculated as the ratio of the actual number of edges to the maximum number of possible edges. The sparsity‐based thresholding strategy guarantees an equal number of edges among participants and in each category of morphological brain networks. A range of sequential sparsity values from 0.02 to 0.4, with an interval of 0.02, was used to threshold each matrix [14].

#### 2.6.2 Network Parameter Calculation

Both the global and nodal topological metrics of MSN were calculated with the NetworkX software package (https://networkx.org). The global metrics encompassed the global efficiency, local efficiency, clustering coefficient, and typical path length. The node‐centrality metrics, including degree centrality, betweenness centrality, and eigenvector centrality, were also calculated. Formulas, usages, and descriptions of these metrics are available in other sources [55]. The AUC for each metric was additionally computed to get a condensed numerical value for subsequent statistical analyses.

### 2.7 Gene Expression Correlation Analysis

#### 2.7.1 Estimation of Regional Gene Expressions

The AUC for each metric was additionally computed to get a condensed numerical value for subsequent statistical studies. The data was processed using the practical pipeline described in the previous study [56]. Specifically, there were six processing steps to obtain a region gene expression matrix: (1) the probe‐to‐gene annotations were updated using the latest available information from the National Center for Biotechnology Information (NCBI) using the Re‐Annotator package [57]; (2) probes that did not exceed background noise intensity in at least 50% of samples across all donors were filtered out; (3) the probe with the highest differential stability (DS) [58] across donors was selected; (4) samples were assigned to the regions of Destrieux atlas in terms of standard deviations [59] (< 2 standard deviations); (5) Expression values for each donor were normalized using a scaled robust sigmoid transform [60] to account for differences between samples and donor‐specific effects on gene expression; and (6) Genes were filtered based on DS to reduce donor‐specific variance, and the top 50% of genes with the highest DS were chosen for the main analysis. Due to the limited availability of right hemisphere data in the AHBA dataset, this study focused exclusively on the left hemisphere to reduce variability among regions. Ultimately, the gene expression matrix (74 regions × 8476 gene expression levels) was created by calculating the transcriptional level of each gene in each brain region of the left hemisphere.

#### 2.7.2 Imaging‐Transcriptomic Analysis

Partial least squares (PLS) regression was used to examine the spatial correlations between neuroimaging indicator differences (posttraining vs. baseline) and transcriptional levels for all 8476 genes. Gene expression data (74 × 8476 matrix) was used as predictor variables; the cortical index (CT and FD) and topological metrics of MSN (degree centrality, betweenness centrality, and eigenvector centrality) difference values were used as response variables. The first component identified in the PLS analysis (PLS1) represented the linear combination of gene expression levels that exhibited the strongest correlation with neuroimaging indicator disparities. A permutation test was performed 5000 times to assess whether the PLS1 component explaining variations in the cortical index was significantly more than what would be anticipated by chance.

Following the acquisition of the PLS weights for each gene, a bootstrapping technique was employed to evaluate the stability of each gene. This involved generating 5000 bootstrap samples. The *Z* score was computed as the ratio of the weight to its bootstrap standard error for each gene. Following a one‐sample *Z* test and subsequent false discovery rate (FDR) correction (*p* < 0.05, *z* > 1.96), two gene lists were generated: one consisting of genes with significantly positive associations with cortical index differences (PLS1+), and another consisting of genes with significantly negative associations with cortical index differences (PLS1−).

#### 2.7.3 Enrichment Analysis

The enrichment analysis for PLS1 genes was conducted using the gene ontology (GO) biological process in the web programm (https://biit.cs.ut.ee/gprofiler/gost) [61]. Two sets of genes (PLS+ and PLS−) were entered into the online platform separately to conduct overrepresentation analysis for Gene Ontology (GO) terms. A multigene list of meta‐analysis was performed to identify the common biological processes shared by two samples. All enrichment pathways obtained were adjusted using the FDR‐corrected threshold for statistical significance at a *p*‐value of less than 0.05.

### 2.8 Statistical Analysis

The JASP software (https://jasp‐stats.org) was utilized for statistical analysis. The Shapiro–Wilk test was employed to assess the normality of numerical data; if the *p* value from the test was greater than 0.05, the data were considered normally distributed. Data that do not meet a normal/Gaussian distribution were analyzed using non‐parametric test. The categorical variables were examined using Chi‐square tests. Two‐sample *t*‐tests were used for independent samples of continuous variables and the rank‐sum tests were used for non‐normally distributed data. The analysis of graph theory metrics involved the use of *t*‐tests, with adjustments made for multiple comparisons. These adjustments included the application of the FDR correction and the false positive correction method [62]; the latter was particularly for node‐specific metrics (using a threshold of *p* < 1/148 = 0.0067).

## 3 Results

### 3.1 Demographic and Cognitive Characteristics

The demographic characteristics, including age (19.139 ± 0.867 vs. 19.222 ± 0.542, *t* = 0.489, *p* = 0.626), sex (23/13 vs. 22/14, ×2 = 0.059, *p* = 0.808), and education level (13.972 ± 0.291 vs. 13.944 ± 0.410, *t* = 0.331, *p* = 0.741), were not significantly different between the WMT group and control group (Table 1).

**TABLE 1:** Demographic characteristics of participants.

	WMT (*n* = 36)	Control (*n* = 36)	*t* (*x* ^2^)	*p*
Sex (female/male)	23/13	22/14	0.059	0.808
Age (years)	19.139 ± 0.867	19.222 ± 0.542	0.489	0.626
Education (years)	13.972 ± 0.291	13.944 ± 0.410	0.331	0.741

**FIGURE 2:**
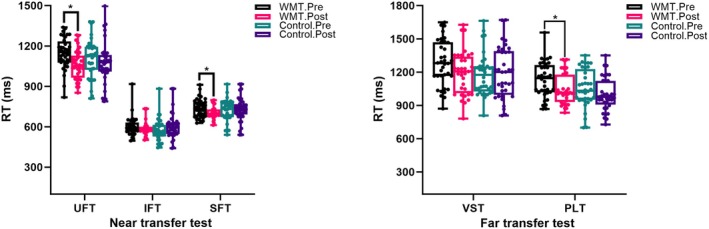
Cognitive assessment comparisons (near‐transfer test and far‐transfer effect) among groups. Control, control group; IFT, inhibition function test; PLT, phonological loop test; RT, response time; SFT, switching function effect; UFT, updating function test; VST, visuospatial test; WMT, working memory training group. FDR correction, *p < 0.05.

Following WMT, several cognitive tests showed significant changes within the WMT cohort (Figure 2). Specifically, the response times in the near‐transfer test, such as updating function (1043.769 ± 147.596 vs. 1149.000 ± 118.288, *t* = 2559, *p* = 0.014, *q* = 0.014) and switching function (693.231 ± 53.257 vs. 740.486 ± 67.091, *t* = 2.281, *p* = 0.027, *q* = 0.014), and far‐transfer test, such as phonological loop test (1004 ± 153.143 vs. 1133 ± 165.504, *t* = 2.367, *p* = 0.022, *q* = 0.044), became more quick in post‐training than in the baseline. But the accuracy of the tests did not show any difference within the group cohort or between the group cohorts.

### 3.2 Cortical Morphology

**FIGURE 3:**
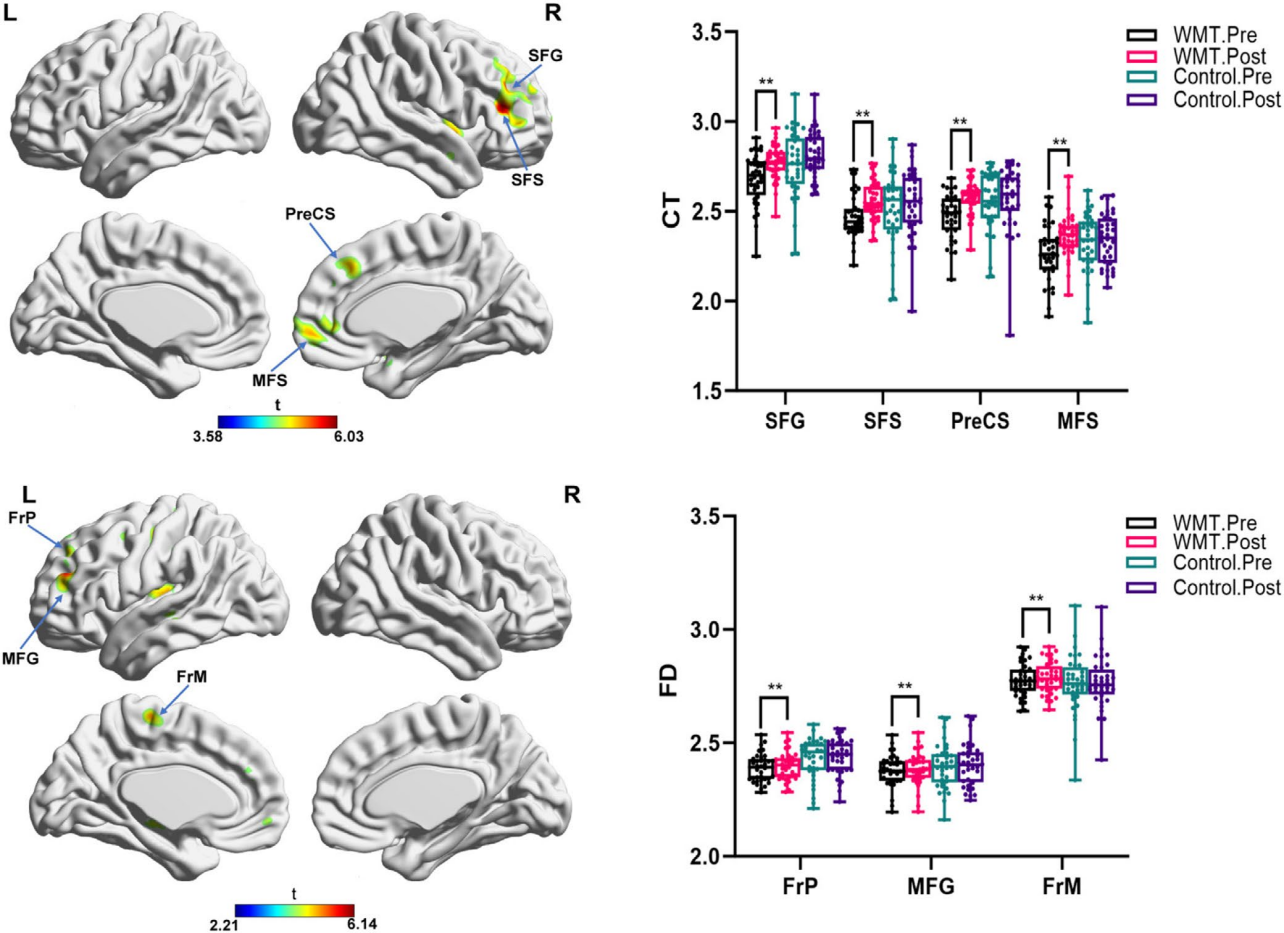
Cortical morphological metrical comparisons of cortical thickness (CT) and fractional dimension (FD) among groups. False positive correction, **p < 0.01. Control, control group; FrM, Frontomargin; FrP, frontopole; MFG, middle frontal gyrus; MFS, middle frontal sulcus; PreCS, precentral sulcus; SFG, superior frontal gyrus; SFS, superior frontal sulcus; WMT, working memory training group.

During the analysis of cortical morphological measurements between different groups, specific cortical metrics such as cortical thickness (CT) and fractal dimension (FD) exhibited noticeable alterations within the WMT cohort (Figure 3). Specifically, the WMT group showed a significant increase in CT in some areas of the prefrontal cortices, such as the right superior frontal gyrus and sulcus, right middle sulcus, and right precentral sulcus, after the training compared to baseline. Likewise, an increase in the FD of specific cortical areas involved the bilateral transverse frontopolar regions, the left frontomarginal area, and the left middle frontal gyrus. But when examining other cortical morphological metrics such as SD and GI, no significant alterations were observed.

### 3.3 Global Topological Metrics of MSN

Building on the established results of the cortical morphological metrics, MSN using CT and FD were further constructed. By employing global indices derived from graph theory, we evaluated several metrics such as global efficiency, local efficiency, clustering coefficient, and characteristic path length (Figure S1). The thorough analysis indicated that there were no notable disparities in these measures, both in pre‐ and post‐assessments within the WMT and control cohort, as well as in comparisons between the pre‐ and post‐assessments of different cohorts.

### 3.4 Nodal Topological Metrics of MSN

**FIGURE 4:**
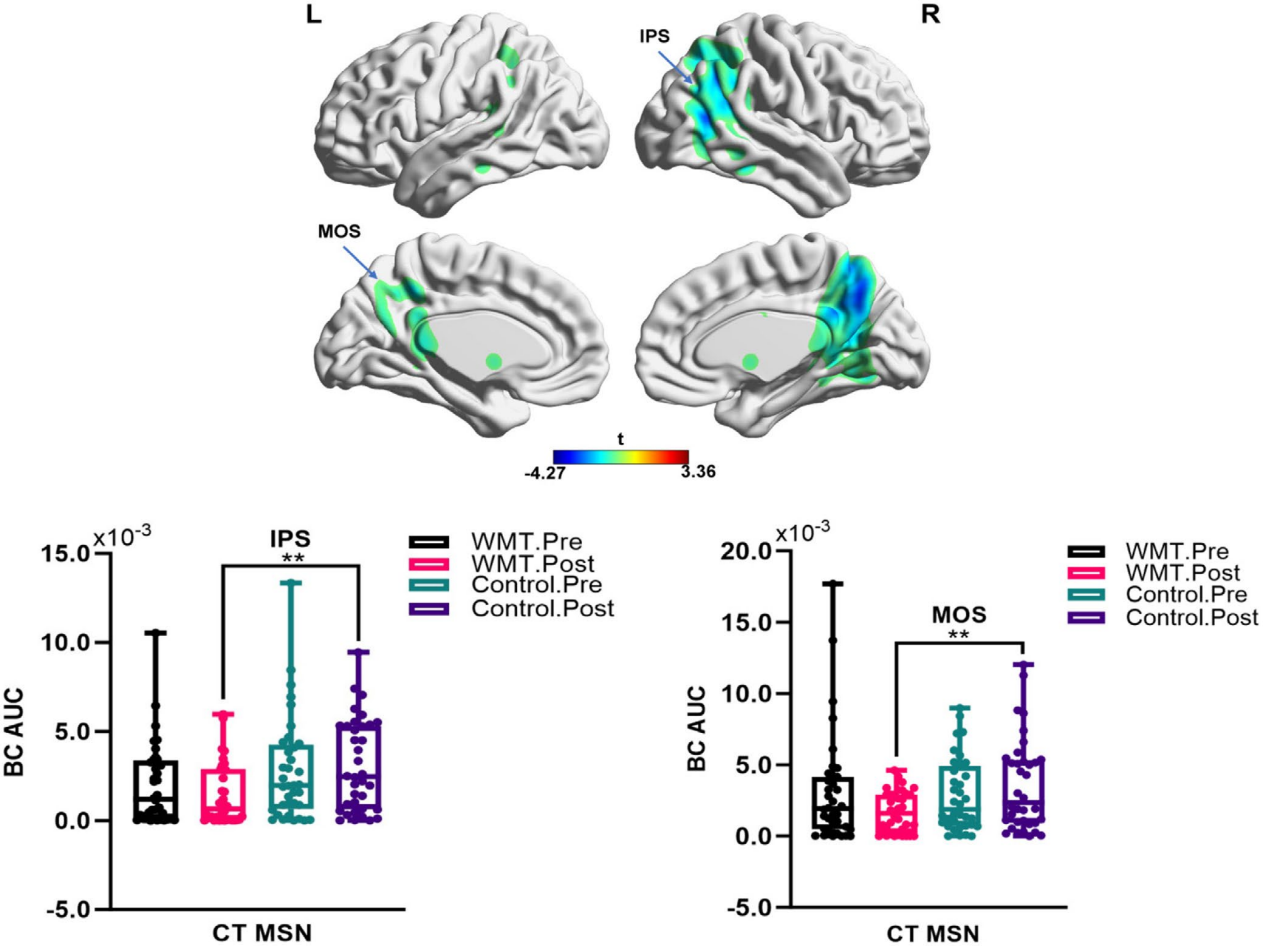
Nodal metric comparisons in the morphometric similarity network (MSN) of cortical thickness (CT) among groups. BC, between centrality; AUC, area of under the curve. False positive correction, **p < 0.01. Control, control group; IPS, intraparietal sulcus; MOS, middle occipital sulcus; WMT, working memory training group.

Nodal topological metrics within the CT MSN uncovered distinct alterations (Figure 4). Significantly, there was a reduction in betweenness centrality in the left middle occipital sulcus and right intraparietal sulcus during the post‐assessments after the working memory training, in comparison to the post‐assessment measurements of the control group.

**FIGURE 5:**
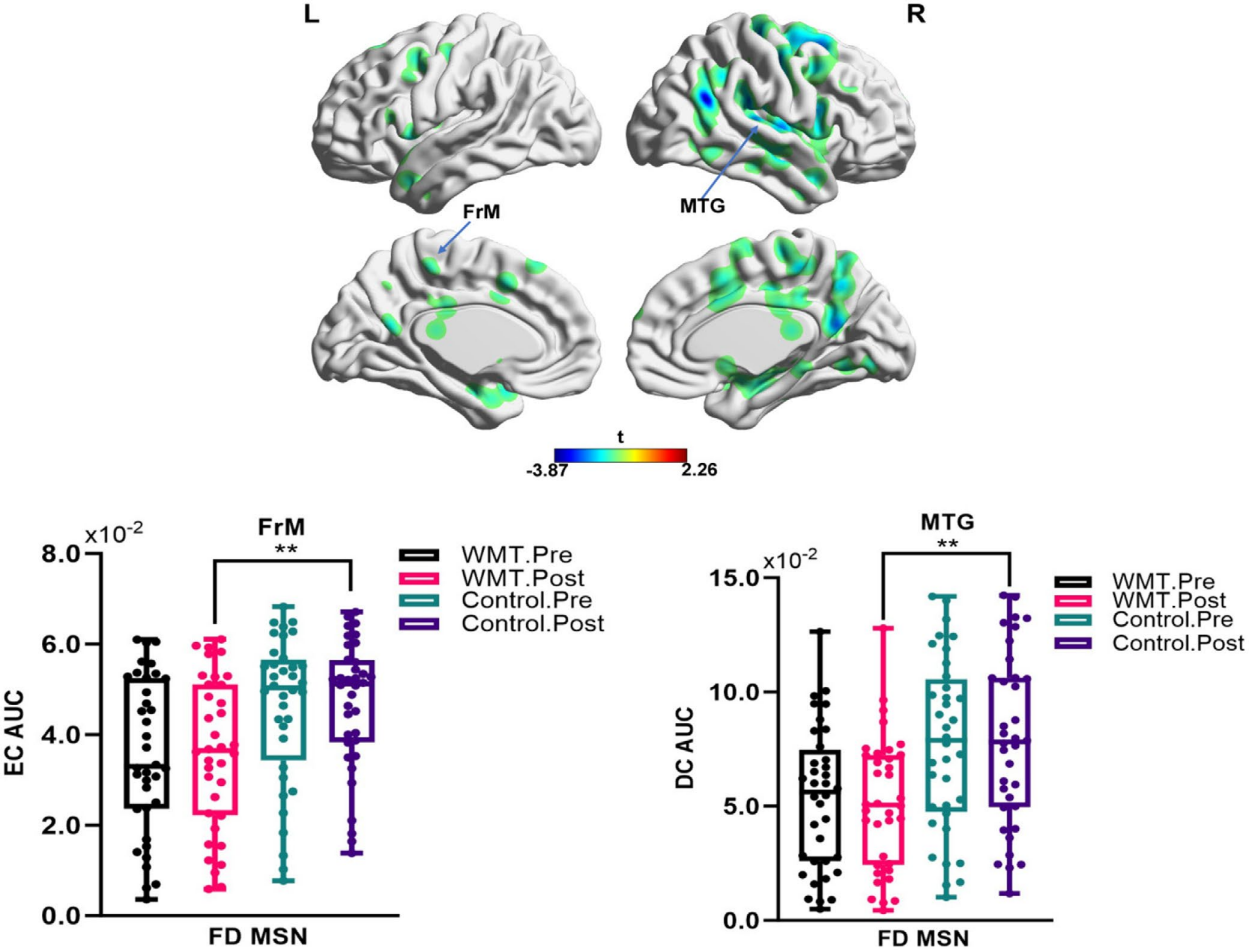
Nodal metric comparisons in morphometric similarity network (MSN) of fractional dimension (FD) among groups. AUC, area of under the curve; Control, control group; DC, degree centrality; EC, eigenvector centrality; FrM, Frontomargin; MTG, middle temporal gyrus; WMT, working memory training groupFalse positive correction, **p < 0.01.

Significant findings were made regarding the left frontomarginal cortex inside the FD MSN, as depicted in Figure 5. After undergoing WMT, the region showed a drop in eigenvector centrality compared to the control group's post‐assessment metrics. In addition, the FD MSN showed a comparable pattern in the right middle temporal gyrus, with a drop in degree centrality observed in the post‐WMT assessment compared to the post‐control assessment (Figure 5).

### 3.5 Biological Process of Gene Enrichment

**FIGURE 6:**
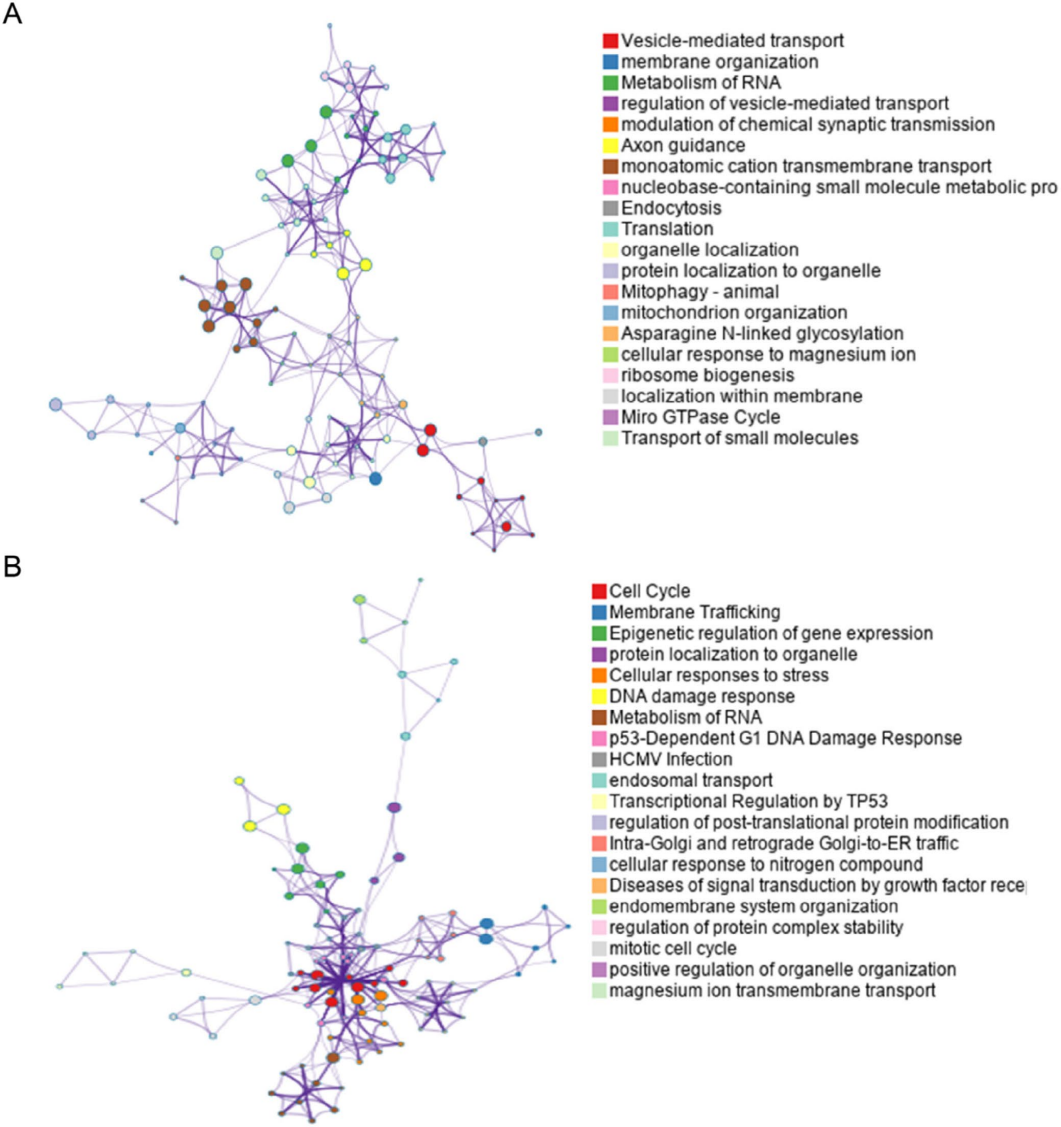
Functional enrichment of gene transcripts. Functional annotation of the biological processes (BP) for PSL + genes (A) and for PLS1‐ genes (B). Each node represents a BP term, and nodes are color‐coded by their functional category, as indicated in the legends. The connections between nodes reflect functional relationships.

The PLS analysis revealed a strong correlation between changes in cortical morphological indices (CT and FD) and gene transcript expression levels, but not topological characteristics. Figure 6 displays the outcomes of functional annotation for two sets of genes associated with cortical morphological index changes.

Specifically, the genes in PLS+ showed a significant enrichment in biological processes including synaptic transmission and neural regulation, vesicle transport and membrane organization, and energy metabolism and mitochondrial function. While the genes in PLS− were mainly associated with biological processes including intracellular transport and membrane trafficking, protein localization and modification, stress responses, and DNA damage repair.

## 4 Discussion

This research assessed the effects of an 8‐week computerized WMT regimen on the cognitive performance and neuroimaging biomarkers, including cortical structure and the topological properties of MSN, in healthy adults. The results from the post‐training assessments demonstrate that the WMT group not only showed improved RTs in these transfer tasks, but significant changes were also observed in the microstructure plasticity of the brain. Considerable alterations were observed in cortical morphology, as evidenced by increases in CT and FD in specific brain regions, as well as nodal topological aspects of MSN in certain areas. Furthermore, the cortical morphological index changes were connected to specific gene expression, which provides a plausible biological rationale for the impact of WMT. These results imply that the training's duration and intensity were adequate to produce detectable neuroplastic alterations. These findings emphasize the capacity of WMT to enhance cognitive function and induce distinct morphological alterations in the brain.

### 4.1 Improvements in Cognitive Functions

WM is essential for performing daily cognitive tasks. Researches indicate that engaging in WMT can enhance both the capacity of working memory and the related cognitive abilities [3, 47]. Updating refers to the ability to substitute outdated information in WM, whereas switching refers to the capacity to smoothly transition between several tasks or modes of thinking. Improved proficiency in these domains indicates that WMT has the potential to enhance cognitive regulation and adaptability. In addition, WM is intricately associated with the understanding and generation of language [63]. There is evidence suggesting that WMT can facilitate verbal ability [64]. This means that an enhanced WM capacity can assist in more efficient processing and comprehension of language information [65]. The findings indicate that WMT has the potential to enhance performance not only in specific working memory tasks but also in broader cognitive domains.

### 4.2 Alterations in Cortical Morphology

The increase in CT after WMT in specific brain regions, including the superior frontal gyrus, superior and middle frontal sulcus, and precentral sulcus, indicates that the brain has the ability to undergo substantial neuroanatomical modifications beyond the developmental maturation period. Specifically, the superior frontal gyrus is a component of the dorsolateral prefrontal cortex, which has a significant impact on executive processes, WM, and the regulation of attention [66, 67, 68]. The superior frontal sulcus plays a role in advanced cognitive functions, such as reward processing, WM, executive function, and decision‐making [69, 70]. The precentral sulcus is a conspicuous groove located right in front of the precentral gyrus. It plays a key role in motor planning, execution, and control [71, 72, 73]. The middle frontal sulcus, situated within the middle frontal gyrus, is a component of the neural network that facilitates WM and executive functions [74, 75]. The observed increases in CT in these regions highlight the brain's extraordinary adaptability and its capacity to adjust and enhance through specific cognitive training.

Similarly, the WMT group exhibited an augmentation in the FD of the cortical surface in the frontopolar cortex, frontomarginal cortex, and middle frontal gyrus compared to the baseline measurements. A higher FD typically signifies a more complex cortical surface, indicating a higher level of neural connectivity and processing capacity. The frontopolar cortex, located at the frontmost section of the frontal lobes, is responsible for higher‐level cognitive processes such as executive control, decision‐making, and WM [76, 77, 78]. The frontomarginal cortex, situated at the rostral margin of the orbital part of the frontal lobe, is primarily associated with executive function, WM, decision‐making, and perspective‐taking [79, 80, 81]. The middle frontal gyrus, a critical structure of the dorsolateral prefrontal cortex, is responsible for WM and attention regulation [82, 83, 84, 85]. The observed increases in FD within these areas indicate that WMT may enhance the complexity and functional capacity of regions involved in executive functions and WM. Together, changes in cortical thickness and complexity might reflect neuroplastic adaptations in response to the cognitive demands of WMT and imply a potential structural basis for improvements in cognitive abilities following WMT. This aligns with existing evidence that adult individuals experience structural brain plasticity during the learning process [86, 87].

Our results indicate that WMT induces localized structural changes in specific regions associated with working memory and executive function rather than widespread structural changes. This aligns with previous literature showing that cognitive training often leads to changes in specific brain regions rather than widespread alterations across the entire brain [66, 71, 74, 77, 85]. For example, a longitudinal study by Nichols et al. found that specific cognitive task training resulted in changes in the microstructure of white matter in specific brain regions, with language suppression control training leading to changes in the left frontotemporal and occipital frontal bundle networks, and visual spatial working memory training leading to changes in the right frontal parietal lobe bundle, but there was almost no overlap in white matter microstructure changes between these two groups [88]. Additionally, studies by Draganski and May have demonstrated that training‐induced neuroplasticity tends to be region‐specific, reflecting the functional demands of the training tasks [89].

In particular, when examining other cortical morphological metrics such as SD and GI, no significant alterations were observed. The observed changes of CT and FD in our results aligns with their roles as dynamic markers of microstructural plasticity [50, 90]. In contrast, SD and GI, although important, are more influenced by stable developmental or genetic factors and are therefore less sensitive to short‐term interventions and may require longer longitudinal sampling [91, 92].

### 4.3 Alterations in Topological Characteristics of MSN

The absence of significant changes in the global indices of topological characteristics, such as global efficiency, local efficiency, clustering coefficient, and characteristic path length, indicates that WMT does not significantly disrupt the overall structural and fractal complexity‐based network organization. While numerous studies have demonstrated localized structural and functional changes associated with WMT, evidence for large‐scale network reorganization remains limited and inconsistent [93, 94]. The absence of significant changes in the global indices may suggest that 8 weeks WMT may primarily induce local rather than global neural adaptations. Melby‐Lervåg et al. concluded that WMT produces narrow, task‐specific neural effects rather than broad cognitive or network‐level changes [90]. Similarly, a 2024 meta‐analysis by Yao et al. found that the neural changes associated with the WM training effect occur in the frontoparietal network and dopamine‐related brain areas. Furthermore, combining the results on shorter (< 10 h) and longer (≥ 10 h) WM training, they demonstrated that the training dosage moderates the neural change of WM training effects [95]. Our findings provide support for this view. Namely, while 8 weeks WMT produces reliable local neural effects, its impact on global networks may require either longer training durations or complementary interventions to manifest detectable changes.

The reduced betweenness centrality in the intraparietal sulcus and middle occipital sulcus suggests a shift in the way that these regions communicate within MSN of CT. The intraparietal sulcus is involved in spatial attention and WM [96, 97], and it plays a crucial role in the integration of sensory information [98, 99]. Additionally, it is essential for tasks that involve visuospatial processing [100, 101]. The middle occipital sulcus plays a central role in visual processing [102] and is crucial for interpreting visual perception [103, 104]. The reduced betweenness centrality observed in the intraparietal and middle occipital sulcus after undergoing WMT suggests a possible restructuring of the brain's network in processing sensory information. This reconfiguration likely indicates a more effective cognitive processing mechanism in these areas, demonstrating the brain's ability to adapt to the cognitive requirements and training effects of WMT.

The frontomarginal cortex, as previously stated, is implicated in higher cognitive processes such as executive function and decision‐making. The middle temporal gyrus is involved in semantic processing, semantic priming, and semantic control [105, 106, 107]. Essentially, the decrease of centrality measures in the frontomarginal cortex and middle temporal gyrus within the MSN of FD after WMT suggests a specific reorganization in brain network dynamics. This reorganization is likely a result of adjustments in cognitive processing, specifically executive function, WM, and language, underpinned by neuroplastic changes. These adjustments highlight the brain's ability to reorganize itself in response to specific cognitive activities, with changes in network dynamics closely matching the cognitive functions of the afflicted regions.

### 4.4 Gene Enrichment for Biological Functions

The PLS analysis revealed distinct sets of genes associated with positive and negative weights (PLS+ and PLS−) in neuroimaging indicator changes (CT and FD) following WMT. These findings suggest that WMT modulates cortical morphological indices through diverse biological pathways, shedding light on the potential neurobiological mechanisms underlying cortical plasticity. Genes with positive weights (PLS+) were primarily enriched in processes related to synaptic transmission and neural regulation, vesicle transport and membrane organization, and energy metabolism and mitochondrial function. These findings align with the hypothesis that synaptic plasticity and enhanced energy metabolism play a central role in cortical reorganization induced by cognitive training. Specifically, synaptic transmission and neural regulation are fundamental processes that support communication between neurons and the remodeling of neuronal network activity [108]. These processes involve neurotransmitter release, plasticity‐related changes, and synaptic pruning. The observed enrichment of synaptic processes in PLS+ genes suggests that complex cognitive training‐induced cortical thickening and complexity are driven by enhanced synaptic efficiency and connectivity [109]. These changes are likely responsible for the observed improvements in cognitive performance. Vesicle transport and membrane organization govern the intracellular trafficking of neurotransmitters, proteins, and organelles [110], facilitating efficient synaptic signaling and maintaining neuronal homeostasis [111]. PLS+ enrichment in these processes highlights the role of intracellular transport in supporting the structural demands of neural plasticity [112]. Energy metabolism and mitochondrial function are vital for meeting the increased energetic demands of neuronal activity during training [113]. Mitochondria not only generate adenosine triphosphate but also regulate calcium signaling and oxidative stress [114, 115], both of which are essential for neural plasticity. The enrichment of energy metabolism‐related processes in PLS+ genes suggests that cortical plasticity changes are supported by heightened metabolic activity [116].

Genes with negative weights (PLS−) were primarily enriched in processes related to intracellular transport and membrane trafficking, protein localization and modification, stress responses, and DNA damage repair. These processes might reflect stress‐induced neural adaptations in response to prolonged cognitive demands. Processes related to intracellular transport and membrane trafficking maintain cellular homeostasis by recycling proteins, organelles, and membrane components [117]. The enrichment of PLS− genes in intracellular transport processes suggests a possible trade‐off between synaptic remodeling and homeostatic mechanisms. Protein localization and modification are critical for ensuring proper cellular function [118], particularly in dynamically adapting environments like those induced by cognitive training. Enrichment of PLS− genes in these processes suggests a compensatory mechanism where protein modifications and accurate localization help maintain cellular function amid training‐induced structural changes. These findings suggest that PLS+ genes reflect cortical plasticity promoted by WMT, while PLS− genes highlight compensatory mechanisms under increased neural demands, together revealing a dynamic balance underlying cortical morphological changes.

### 4.5 Clinical Implications

While the study focuses on healthy adults, the findings may have implications for clinical populations, such as individuals with aging‐related cognitive decline or neurodegenerative disorders like Alzheimer's disease. Working memory deficits are often found in aging individuals, especially in those with mild cognitive impairment or early stages of Alzheimer's disease [119, 120]. Normal aging is associated with the structural and functional brain changes that can affect cognitive domains including working memory [121]. For example, the increases in prefrontal cortical thickness and fractal dimension observed in the present study could suggest that WMT might help mitigate age‐related cortical thinning, which aligns with the findings that cognitive training can mitigate age‐associated structural brain changes in the elderly [122]. However, studies on computerized cognitive training programs in cognitive impairment individuals showed mixed results [123]. Further research is needed to determine whether these findings generalize to clinical populations.

### 4.6 Limitations

Firstly, we acknowledge that there are potential confounding factors (e.g., baseline cognitive ability, lifestyle factors), and future research should consider controlling for these factors to ensure that the observed effects are entirely attributable to WMT. Secondly, the PLS regression analysis identifies the correlation between morphological network node changes and AHBA gene expression. However, this represents only inferred correlations. In future research, we will consider validating our current findings using in vivo models, such as qPCR. Moreover, we acknowledge the limitation of focusing solely on the left hemisphere due to AHBA data constraints.

## 5 Conclusion

In summary, an 8‐week WMT program caused notable changes in the cortical microstructure and the topological characteristics of key nodes in the MSN in healthy individuals. These changes occurred in regions of the brain that are important for WM and gene transcriptional expression. These findings suggest that WMT has the potential to induce targeted neuroplasticity in the brain and provide integrative understanding of the underlying biological neural mechanisms.

## Author Contributions

Tian Zhang performed the initial statistical analysis and wrote the first draft of the manuscript. Yuntao Gao and Yijun Li produced and organized the datasets. Lin Wu and Xinxin Lin conducted imaging data processing, and Yihou Hou assisted with preprocessing and visual checking. Wei He and Yuanqiang oversaw participant recruitment. Jun Jiang and Yuanjun Xie assisted in additional analyses and writing of the manuscript. Peng Fang and Yuanjun Xie provided feedback on the study design during the study implementation. All authors have contributed to and approved the final manuscript.

## Conflicts of Interest

The authors declare no conflicts of interest.

## Supporting information


**Figure S1:** Global topological metrics comparisons in the morphometric similarity network (MSN) of cortical thickness (CT) and fractional dimension (FD) among groups.

## Data Availability

The data that support the findings of this study are available from the corresponding author upon reasonable request.
