# Supplementary figures and images for: Neural Plasticity Induced by Working Memory Training: Insights From Cortical Microstructure and Transcriptional Profiles
Source: CNS Neurosci Ther. 2025 Jul 30;31(8):e70479. doi: 10.1111/cns.70479 (PMC12311235; doi:10.1111/cns.70479)

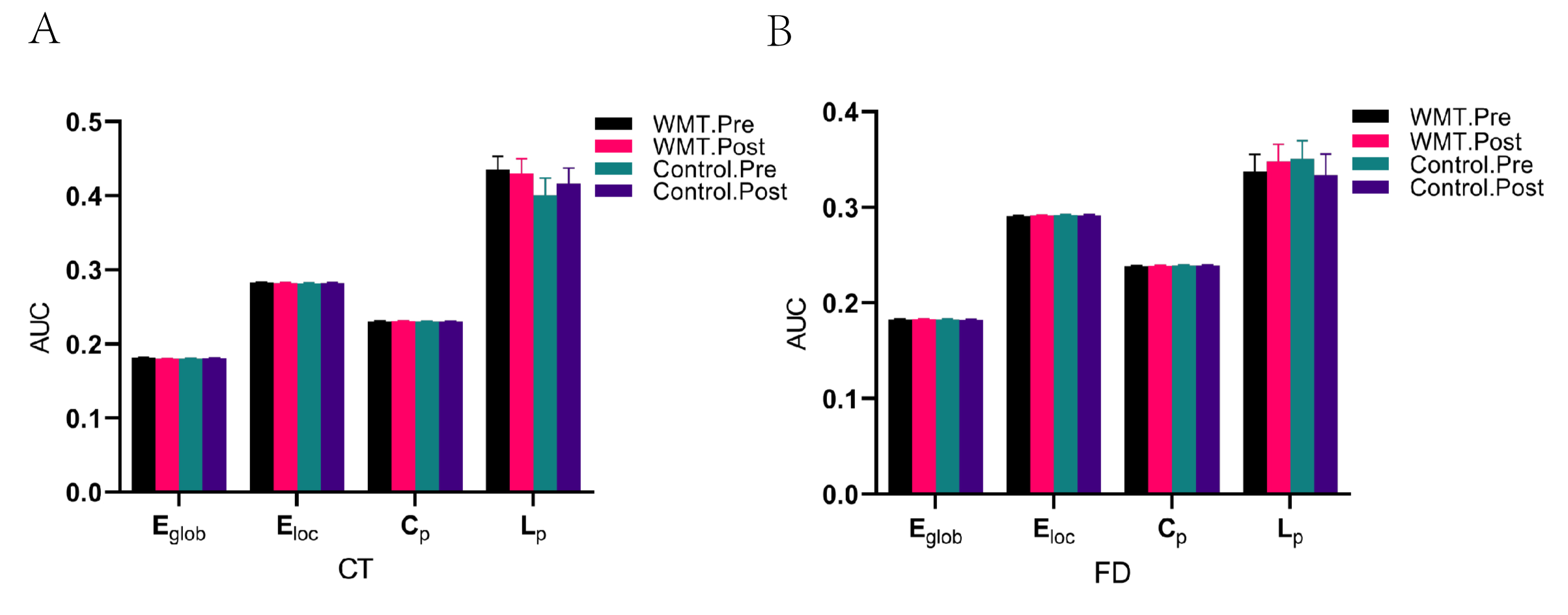

Supplement: Supplementary file 1 — Figure S1: Global topological metrics comparisons in the morphometric similarity network (MSN) of cortical thickness (CT) and fractional dimension (FD) among groups. [file CNS-31-e70479-s001.tif]
